# Discovery of a new mammal species (Soricidae: Eulipotyphla) from Narcondam volcanic island, India

**DOI:** 10.1038/s41598-021-88859-4

**Published:** 2021-05-03

**Authors:** Manokaran Kamalakannan, Chandrakasan Sivaperuman, Shantanu Kundu, Govindarasu Gokulakrishnan, Chinnadurai Venkatraman, Kailash Chandra

**Affiliations:** 1 Mammal and Osteology Section, Zoological Survey of India, Kolkata, 700053 India; 2 Molecular Systematics Division, Centre for DNA Taxonomy, Zoological Survey of India, Kolkata, 700053 India; 3 Andaman and Nicobar Regional Centre, Zoological Survey of India, Port Blair, 744102 India

**Keywords:** DNA, Zoology, Sequencing, Sequencing, Classification and taxonomy, Phylogeny, Sequence annotation, Molecular evolution, Phylogenetics, Speciation, Taxonomy

## Abstract

We discovered a new *Crocidura* species of shrew (Soricidae: Eulipotyphla) from Narcondam Island, India by using both morphological and molecular approaches. The new species, *Crocidura narcondamica* sp. nov. is of medium size (head and body lengths) and has a distinct external morphology (darker grey dense fur with a thick, darker tail) and craniodental characters (braincase is rounded and elevated with weak lambdoidal ridges) in comparison to other close congeners. This is the first discovery of a shrew from this volcanic island and increases the total number of *Crocidura* species catalogued in the Indian checklist of mammals to 12. The newly discovered species shows substantial genetic distances (12.02% to 16.61%) to other *Crocidura* species known from the Indian mainland, the Andaman and Nicobar Archipelago, Myanmar, and from Sumatra. Both Maximum-Likelihood and Bayesian phylogenetic inferences, based on mitochondrial (cytochrome b) gene sequences showed distinct clustering of all included soricid species and exhibit congruence with the previous evolutionary hypothesis on this mammalian group. The present phylogenetic analyses also furnished the evolutionary placement of the newly discovered species within the genus *Crocidura*.

## Introduction

The terrestrial, insectivorous mammalian species in the genus *Crocidura* Wagler 1832 (subfamily Crocidurinae of the family Soricidae, order Eulipotyphla) are commonly referred to as white-toothed shrews^1^. *Crocidura* is a widespread and speciose genus with 198 species occurring in Africa, Europe, and Asia^2,3^, making it the most species-rich genus of mammals^4,5^. It is characterized by a small to medium-sized body (head and body length 35–100 mm) with usually short dense grey fur, a first-unicuspid tooth which is large, protrudes forward and is hooked, with a small cusp present behind the main cusp, unpigmented teeth, and no zygomatic arches^6^. The genus *Crocidura* (three upper unicuspids) can be differentiated from the nearest genus *Suncus* (four upper unicuspids) through dental formula^1,7^ (Supplementary Fig. S1). So far, 20 species of *Crocidura* are known from the Indian mainland, the Andaman and Nicobar (AN) Archipelago, Myanmar, and the Sundaic continental shelf island of Sumatra^8^ (Supplementary Table S1). Although these regions house members of 10 genera (*Anourosorex*, *Blarinella*, *Chimarrogale*, *Crocidura*, *Episoriculus*, *Feroculus*, *Nectogale*, *Sorex*, *Soriculus*, and *Suncus*) of the family Soricidae^2,3^, the AN Archipelago is known to house only a single genus *Crocidura*, with four species, namely the Andaman shrew *C. andamanensis*, the Andaman spiny shrew *C. hispida*, Jenkin’s shrew *C. jenkinsi*, and the Nicobar shrew *C. nicobarica*^9^.

In the first record of the genus *Crocidura* from India Miller (1912)^10^ described *C. andamanensis* and *C. nicobarica*, each based on a single specimen collected on the South Andaman Island and Great Nicobar Island, respectively. Subsequently, again based upon a single specimen Thomas^11^ described *C. hispida* from the northern middle Andaman Island and Chakraborty^12^ described *C. jenkinsi* from Mt. Harriet National Park, South Andaman Island^4^.

**Figure 1:**
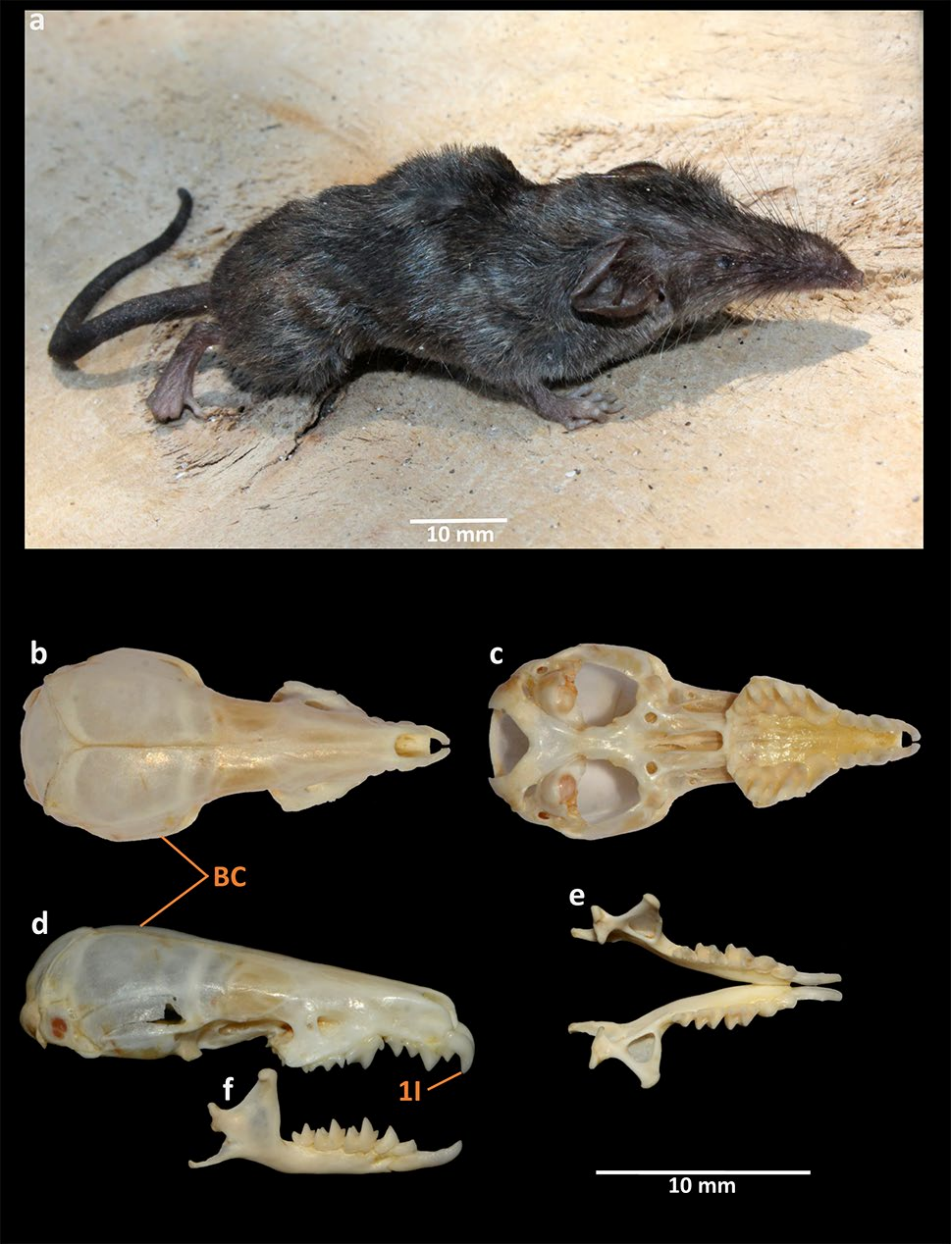
*Crocidura narcondamica* sp. nov. (holotype ZSI-29313, female). (**a**) Dorso-lateral view of female (adult alive). Views of the cranium (**b**) dorsal, (**c**) ventral, and (**d**) lateral. Views of the mandible (**e**) lateral and (**f**) occlusal (*BC* braincase, *I1* first incisor/first unicuspid). The photographs were captured by the first (**b**–**f**) and fourth (**a**) authors using a Nikon D7000 camera and edited manually in Adobe Photoshop CS 8.0.

Due to their secretive behaviour and conservative external morphological characters, shrews are regarded as the least studied mammalian group^5,13^. Consequently, in the last two decades between 2000 and 2020, a total of 24 *Crocidura* species have been newly discovered throughout the world, of which 15 species were discovered in the Indo-Malayan region and Sundaland, especially from the continental islands^2,5,8,14–23^. Molecular studies were also consecutively used to discriminate shrew species, detect cryptic diversity, or study phylogenetic evolution, biogeographic origin and radiation, and phylogeography^8,24–43^. Only a single study aimed to assess the genetic signature of two endemic species, *C. andamanensis* and *C. nicobarica* from the AN Archipelago^44^. Therefore, an understanding of the species diversity on this group of islands remains incomplete. The present study is based on the assumption that a few hitherto unreported shrew species exist beyond the known biogeographic distribution of the group in the AN Archipelago, which warrants further investigation through integrative approaches. We performed both morphological and molecular assessments to confirm a new shrew species from the volcanic Narcondam Island in the AN Archipelago, which is herein described as *Crocidura narcondamica* sp. nov. (Fig. 1). The newly discovered species is validated by a morphometric and molecular comparison with 13 and 15 species, respectively, distributed in the AN Archipelago, on the mainland of India, and the close biogeographic realms of Myanmar and Sumatra. We estimated the genetic divergence from related species and performed phylogenetic analyses to corroborate the taxonomic identity and evolutionary relationships of this novel species.

## Materials and methods

### Study area

**Figure 2:**
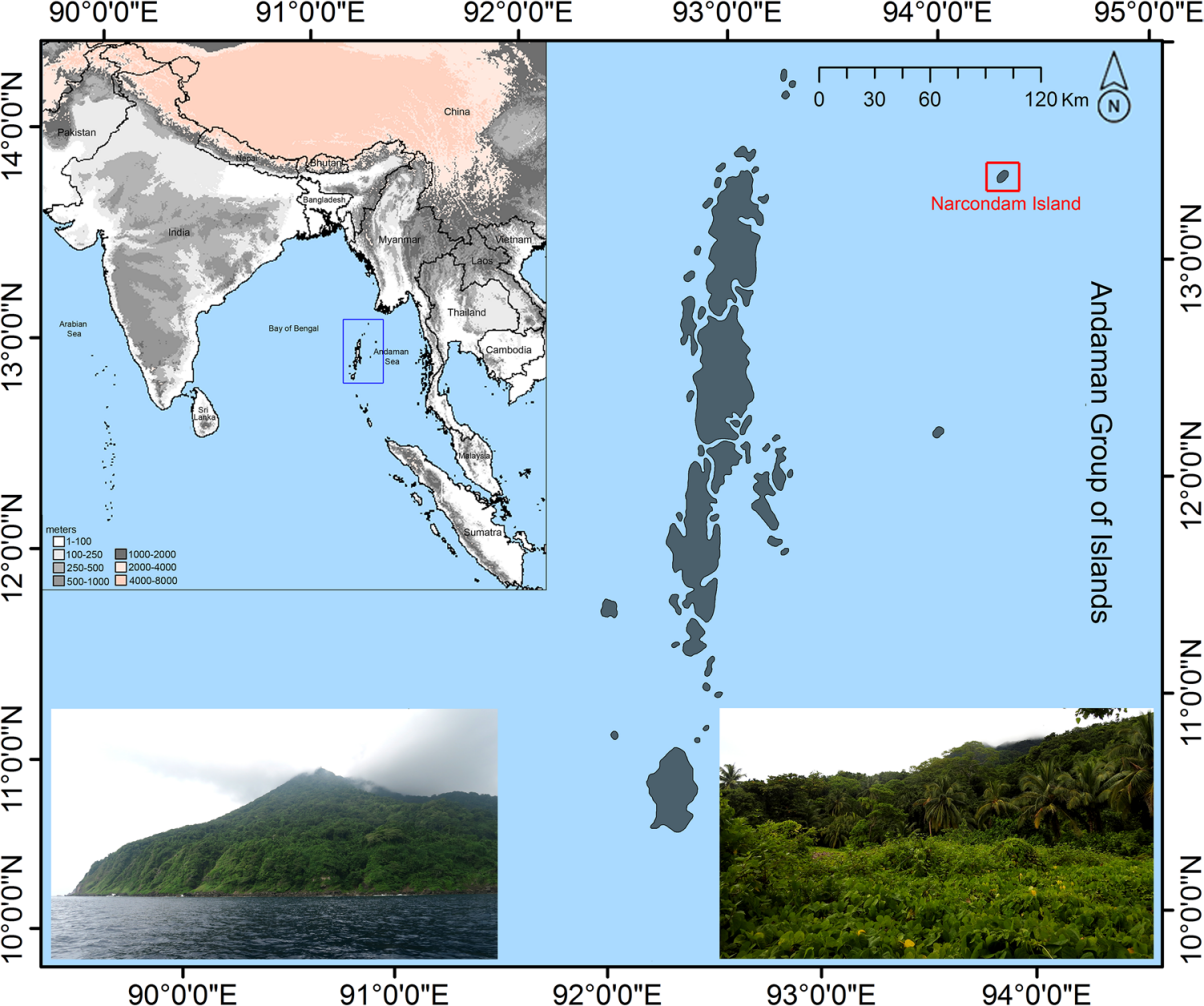
Map showing the Narcondam Island (marked by a red colour box) in the Andaman group of Island, India (marked by a blue colour box) along with the habitat where the new species was encountered. Map prepared using QGIS 2.6.1 (http://www.qgis.org) and edited manually in Adobe Photoshop CS 8.0. The habitat photographs were captured by the fourth author using a Nikon D7000 camera and edited manually in Adobe Photoshop CS 8.0.

Narcondam Island (13.45° N 94.27° E; Fig. 2) is located about 130 km east of North Andaman, and about 446 km off the west coast of Myanmar^45–47^. The island covers an area of 6.8 km^2^ and the highest peak (volcanic cone) is 710 m above sea level; however, the base lies approximately 1500 m beneath the sea^45^. This isolated island is part of a volcanic arc that continues northward from Sumatra to Myanmar^48^. The climatic condition of this small, conical island can be defined as a humid, tropical, and coastal. The island is thickly vegetated, bordered by cliffs on the southern side and crested by three peaks. The forest types can broadly be categorized as three zones: wet evergreen on the slopes and highest zones of the volcano, moist deciduous or semi-evergreen at lower elevations, and littoral forest along the coastline^47^.

### Ethics statement, sampling, and morphological examination

**Figure 3:**
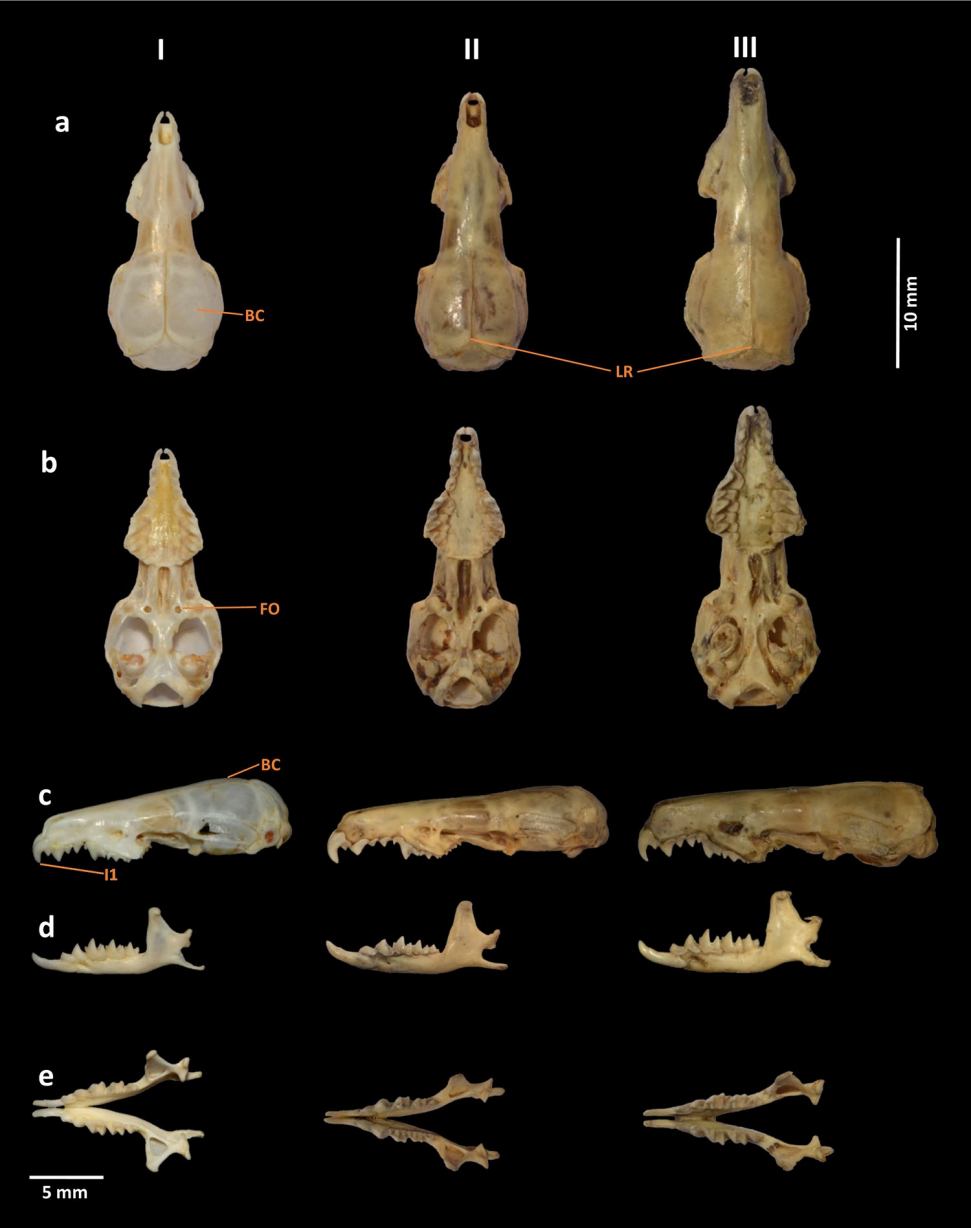
Cranium and mandible of (I) *C. narcondamica* sp. nov. (holotype, ZSI-28000) (II) *C. attenuata* (ZSI-16129) and (III) *C. jenkinsi* (ZSI-19860). From top to bottom, (**a**) dorsal, (**b**) ventral, (**c**) lateral views of the cranium, and (**d**) lateral and (**e**) occlusal views of the mandible. Distinct morphological features are labelled as *BC* brain case, *LR* lambdoidal ridge, *FO* foramen ovale, and *I1* first incisor/first unicuspid. The photographs were captured by the first author using a Nikon D7000 camera and edited manually in Adobe Photoshop CS 8.0.

To conduct the field survey and sampling, prior permission was acquired from the office of the Principal Chief Conservator of Forests (Wildlife), Andaman and Nicobar Islands, Port Blair (Letter no. CWLW/WL/24/339, dated 04 February 2020). The experimental protocols were approved by the Zoological Survey of India and were carried out in accordance with relevant guidelines in compliance with the ARRIVE 2.0. guidelines (https://www.arriveguidelines.org)^49^. Two individuals of this unique shrew species were captured from the same location (13° 27.290′ N 94° 16.436′ E) by the pitfall method. Their external measurements were taken in the field and include head and body length (HB), tail length (TL), ear length (E), and hindfoot length including claw (HF). Photographs of fresh specimens were taken by the fourth author in the field. The collected specimens were preserved in 70% molecular grade ethanol for further investigation. Skulls were extracted later, cleaned, and prepared in the museum (Fig. 1a–e) and deposited in the National Zoological Collections (NZC-Mammal and Osteology section) of the Zoological Survey of India (ZSI), Kolkata, India, under the registration numbers 29313 and 29314. The nomenclature of external and craniodental characters follows Hutterer et al.^22^. Field methods followed the guidelines approved by the American Society of Mammalogists^50^. The craniodental measurements were taken by the first author with a digital caliper accurate to the nearest 0.01 mm. The measurements include condylo-incisive length (CIL), braincase height (BH), rostrum width (RW), hard palatine length (PL), maxillary breadth (MB), least interorbital breadth (LIOB), braincase breadth (BB), upper toothrow length (UTR), maximum breadth across the outer anterobuccal margins of the parastyles of the second upper molars (M2-M2), length of the anterior tip of the fourth premolar (P4) to posterior border of M3 (P4-M3), braincase length (BL), postglenoid width (PGL), the maximum length from the anterior face of the first upper incisor to the posterior margin of the third unicuspid (I-UN3), length of 1st upper incisor (in lateral view) from tip to upper margin of cingulum (LI1), mandibular toothrow length (MTR), length of mandible from the tip of incisor to the posterior edge of the condyle (ML), length of lower molar series (m1-m3), length of 1^st^ lower incisor from tip to posterior margin of the cingulum (Li1), and height of the coronoid process (COR) (Table 1, Supplementary Table S2). Only adult specimens were included, as determined by fully erupted molars and fused basioccipital suture^5,51^. Photographs of cranial and dental views were taken by the first author using a Nikon D7000 camera. The collected specimens of the possible new species of shrew were compared with the morphometric data of 13 congeners reported from the AN Archipelago, the mainland of India and from Myanmar (Supplementary Table S2). The external and craniodental characters were also compared with the archival specimens of *C. attenuata* and *C. jenkinsi* available in the NZC of ZSI, Kolkata (Fig. 3, Supplementary Figs. S2, S3).

**Table 1:** External and craniodental measurements of the examined specimens of *C. narcondamica* (holotype and paratype) from Narcondam Island, India.

Variable	*C. narcondamica* (Holotype, ZSI 29313)	*C. narcondamica* (Paratype, ZSI 29314)
HB	67	63
TL	58.5	55.6
E	6.4	6
HF	13.4	12.4
CIL	19.6	18.9
BH	4.4	4
RW	1.9	1.7
PL	7.4	7
MB	5.8	5.1
LIOB	3	2.8
BB	8.7	8
UTR	8.8	7.9
M2-M2	5.4	5
P4-M3	4.9	4.2
BL	7.3	6.8
PGL	5.7	5.2
I-UN3	3.9	3.7
LI1	2	2
MTR	7.7	7
ML	11.6	10.8
m1-m3	3.5	3
Li1	3.2	3
COR	4.5	4.1

For trait abbreviations (columns) see “Materials and methods” section. All measurements are in millimetres (n = 2).

### Comparative materials

Morphometric measurements of other known congeners distributed in the AN Archipelago, the mainland of India and in Myanmar were also acquired from published literature^5,10,52–54^ (Supplementary Table S2). The morphology and craniodental measurements along with other collateral information of the museum and other comparative species are given in Supplementary Table S2.

### DNA extraction, PCR amplification, and sequencing

The genomic DNA was extracted from both the holotype and the paratype specimen by the standard phenol–chloroform isoamyl alcohol method^55^. The extracted DNA was visualized through 1% agarose gel electrophoresis. The published primer pair (mcb 398: 5′-TACCATGAGGACAAATATCATTCTG-3′ and mcb 869: 5′-CCTCCTAGTTTGTTAGGGATTGATCG-3′)^56^ was used to amplify the widely applied mitochondrial Cytochrome b (mtCytb) gene segment for the identification of shrew species^7,57^. The 25 ml PCR mixture comprises 10 pmol of each primer, 20 ng of DNA template, 1X PCR buffer, 1.0–1.5 mM of MgCl2, 0.25 mM of each dNTPs, and 1 U of Platinum Taq DNA Polymerase High fidelity (Invitrogen). The PCR reaction was performed in Veriti Thermal Cycler (Applied Biosystems) with the published thermal profile. The PCR products were purified using a QIAquick Gel Extraction Kit (QIAGEN) with standard protocol. The cycle sequencing was executed by using BigDye Terminator ver. 3.1 Cycle Sequencing Kit (Applied Biosystems) and 3.2 pmol of each primer on Veriti Thermal Cycler. The products were cleaned by BigDye X-terminator kit (Applied Biosystems) with standard protocol and subsequently bidirectional sequenced by the 48 capillary 3730 Genetic Analyzer (Applied Biosystems).

### Sequence quality control and dataset preparation

The study obtained both forward and reverse chromatograms from the holotype and paratype samples. The noisy parts of each chromatogram were trimmed at both ends and quality value (> 40) was used to screen for making the consensus sequences through the SeqScanner Version 1.0 (Applied Biosystems). The sequences were translated through the online platform ORF finder (http://www.ncbi.nlm.nih.gov/gorf/gorf.html) to validate the sequence by comparison to the vertebrate consensus. The annotated sequences were contributed to GenBank. A total of 54 mtCytb sequences were acquired from GenBank (Supplementary Table S3). A total of 58 sequences were aligned by ClustalX software^58^ to form a combined dataset (473 bp) for further genetic distance and phylogenetic analysis. The sequences (accession no. KP061993 and KP062007) of *Crocidura monax* endemic to Tanzania, East Africa were used as an out-group in the present phylogenetic analyses.

### Genetic distance and phylogenetic analysis

The Kimura-2-parameter (K2P) genetic distance was calculated in MEGAX^59^. The most suitable model for the present dataset was estimated by using JModelTest v2 with the lowest BIC (Bayesian Information Criterion) score^60^. The maximum-Likelihood (ML) phylogenetic tree was constructed using the IQ-Tree web server (http://iqtree.cibiv.univie.ac.at.) with General Time Reversible (GTR) model including a proportion of invariable sites (+ I) and rate of variation across sites and 1000 bootstrap support^61^. The Bayesian (BA) tree was constructed in Mr. Bayes 3.1.2 by selecting nst = 6 for the GTR + G + I with one cold and three hot chains of metropolis-coupled Markov Chain Monte Carlo (MCMC); it was run for 1,000,000 generations with 25% burn-in with trees saving at every 100 generations^62^. The MCMC analysis was used to generate the convergence metrics, until the standard deviation (SD) of split frequencies attained to 0.01 and the potential scale reduction factor (PSRF) for all parameters neared 1.0. The web-based iTOL tool (https://itol.embl.de/) was used for better illustration of the BA phylogenetic tree^63^.

## Results

Mammalia Linnaeus, 1758

Eulipotyphla Waddell et al., 1999

Soricidae G. Fischer, 1814

Crocidurinae Milne-Edwards, 1872

*Crocidura* Wagler 1832

*Crocidura narcondamica* sp. nov.

### Etymology and nomenclatural acts

The new species is named for the type locality, Narcondam Island, where the type specimens were collected. The specific epithet is feminine latinized adjective. Taxonomic nomenclature published in this article follows the amended International Code of Zoological Nomenclature (ICZN, version effective from 1 January 2012). The ZooBank LSID (Life Science Identifier) for this publication can be accessed through urn:lsid:zoobank.org:pub:7EDEF162-C85B-499B-A855-D26E6EB9763F. Suggested common name: Narcondam shrew.

### Type specimens

#### Holotype

Adult female, ZSI 29313, collected at Narcondam Island (13° 27.290′ N, 94° 16.436′ E), Andaman and Nicobar Archipelago, in the Bay of Bengal, India (Fig. 2); 11 m elevation; collected by G. Gokulakrishnan on April 17, 2020. The specimen consists of a fluid-preserved carcass and a cleaned skull. Paratype: Locality and collector same as holotype. Adult male, ZSI 29314. The specimen consists of a fluid-preserved skin and cleaned skull. Skull extracted by the first author.

### Distribution and habitat

The new species is presently known only from its type locality, Narcondam Island, Andaman and Nicobar Archipelago, in the Bay of Bengal, India. It was collected from a littoral forest along the coastline at 11 m elevation. No anthropogenic disturbances were observed in the habitat except for a security post (Fig. 2).

### Diagnosis and description

The new species *C. narcondamica* sp. nov. (Fig. 1) is assigned to the genus *Crocidura* by the presence of three upper unicuspids and clearly distinguished from all other AN Archipelago shrews by its body size and tail length, which are considerably shorter (Supplementary Table S2). The new species possesses a darker-grey dense fur dorsally and a thick and darker tail (Fig. 1a), whereas the species known from the AN Archipelago possesses a different dorsal pelage and tail; *C. jenkinsi* (Supplementary Fig. S3a) and *C. hispida* have a spiny dorsal fur with a slender tail, *C. andamanensis* has a bluish-grey dorsal fur washed with brown and a darker brown tail, *C. nicobarica* has a bristly sooty brown dorsal fur with a slender tail, and *C. attenuata* has a soft brownish-grey dorsal fur with a slender tail^2,51^ (Supplementary Fig. S2a).

The new species also differs from other congeners occurring in the mainland of India, and in Myanmar (Table 1, Supplementary Table S2). The head and body length of the new species (holotype: 67 and paratype: 63 mm) is considerably smaller, than *C. fuliginosa* and *C. pullata*, but overlapping with other species such as, *C. attenuata* (60–89 mm), *C. cranbrooki* (65–86 mm), *C. horsfieldii* (49–71 mm), *C. indochinensis* (53–71 mm), *C. pergrisea* (65–86 mm), *C. rapax* (56–70 mm) and *C. vorax* (54–90 mm). However, the tail length (TL) of the new species (58.5 and 55.6 mm) is longer than *C. horsfieldii* (30–48 mm), *C. indochinensis* (40–50 mm), *C. pergrisea* (39–53 mm), *C. pullata* (37–51 mm), *C. rapax* (38–47 mm) and *C. vorax* (41–51 mm), and shorter than *C. cranbrooki* (65–88 mm), *C. fuliginosa* (62–89 mm). Although the HB and TL of the newly discovered species overlap with *C. attenuata*, the morphological characters are significantly different (soft brownish-grey dorsal pelage with a brownish slender tail; Supplementary Fig S2a).

The length of the hindfoot of *C. narcondamica* sp. nov. (holotype: 13.4 and paratype: 12.4 mm) also differs from three species, *C. pullata* (14–16 mm), *C. cranbrooki* (14–16 mm), and *C. fuliginosa* (15–19 mm), which are distributed in India and Myanmar. However, the rest of the congeners known from the same biogeographic region show an overlapping length of the hindfoot (Supplementary Table S2).

The new species was further examined and compared with the closest congener *C. attenuata* (Fig. 3, Supplementary Fig. S2). The braincase (BC) of *C. narcondamica* sp. nov. is rounded and elevated (Fig. 1b,d), with weaker lambdoidal ridges (LR; Fig. 1b) than in *C. attenuata* (slightly flattened with developed LR; Supplementary Fig. S2c,e). The foramen ovale (FO) is more prominent than in *C. attenuata* (Fig. 3b). In *C. narcondamica* sp. nov., the condylobasal length (holotype: 19.6 and paratype: 18.9 mm), palatal length (7.4 and 7 mm), upper toothrow (8.8 and 7.9 mm), maxillary toothrow (7.7 and 7 mm) and mandible length (11.6 and 10.8 mm) are significantly either higher or lower than in other congeners (Supplementary Table S2). The first incisor (I1) of *C. narcondamica* sp. nov. is less sharp and slightly protruded from the rostrum, than in *C. attenuata* (Figs. 1d, 3c).

### Molecular identification and phylogenetic interpretation

**Figure 4:**
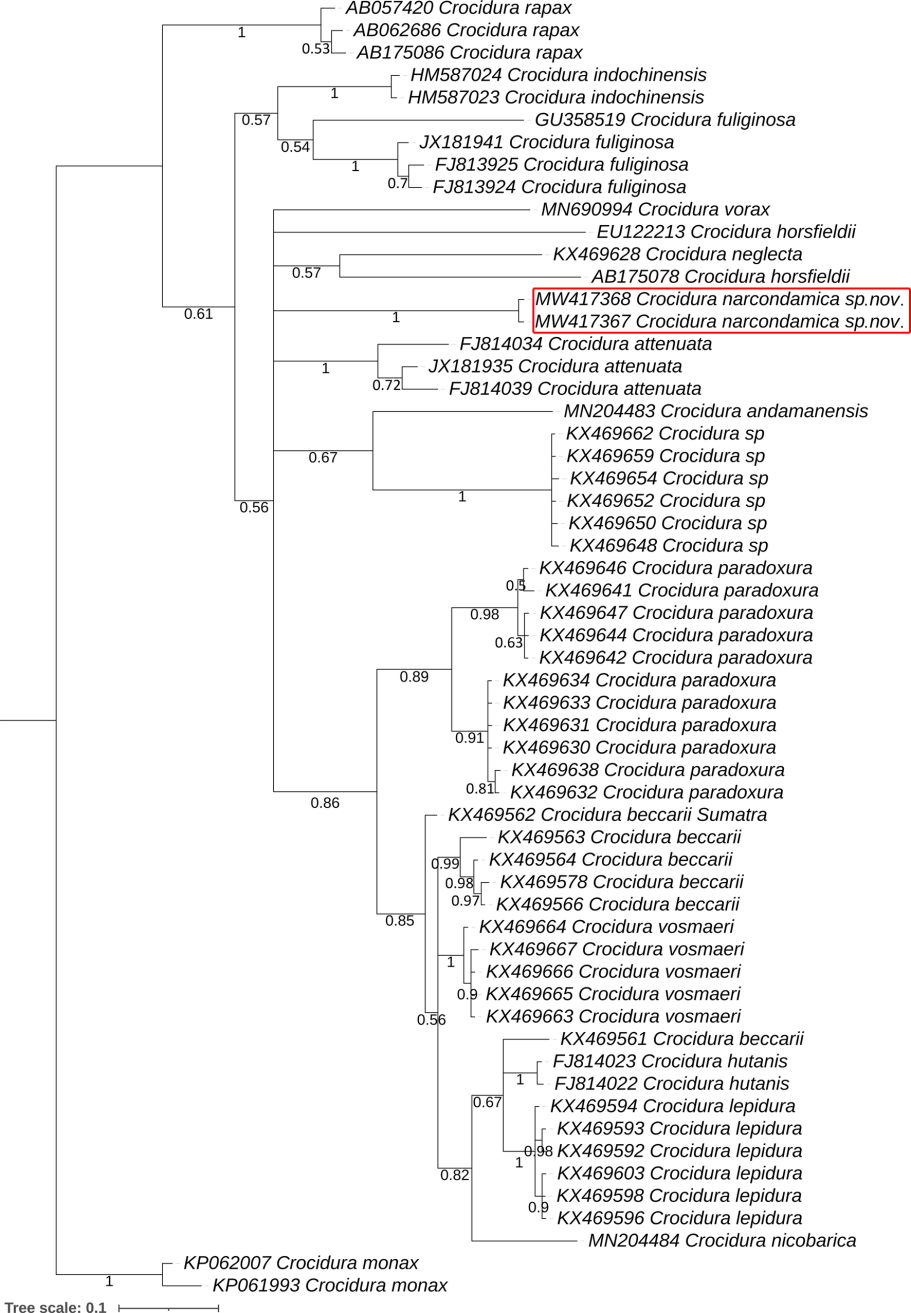
The Bayesian phylogenetic analysis of the mitochondrial Cytb gene depicted a distinct clustering of *C. narcondamica* sp. nov. in comparison with other *Crocidura* species distributed in the AN Archipelago, the mainland of India, Myanmar, and in Sumatra. The posterior probability supports are noted with each node. The GenBank accession numbers and species name are marked as per clade pattern. The distinct clade of the new species is marked by a red colour box. The figure was prepared in web-based iTOL tool (https://itol.embl.de/) and edited manually in Adobe Photoshop CS 8.0.

Partial mitochondrial mtCytb gene sequences (Accession Nos. MW417367 and MW417368) were generated from both the holotype and paratype of the new species and submitted to GenBank. The BLAST search results showed a 90% similarity with the available sequence of a specimen *Crocidura* sp. (MN691031) collected from Xizang, China. The next closest results of the similarity search revealed a 89.57% similarity with a specimen of *Crocidura* sp. (MN691019) collected from Yunnan, China and with *C. attenuata* (MK765768) collected from Jiangxi, China. The present dataset of 21 *Crocidura* species, including the new one shows an overall mean inter-species genetic distance of 11.7%. The new species revealed a substantial mean genetic distance (12.02% to 16.61%) to other *Crocidura* species (Supplementary Table S4). The new species is genetically distant to *C. andamanensis* (16.61%) and *C. nicobarica* (15.09%) distributed in the same group of islands, AN archipelago. The new species also genetically distant (12.02% to 16.57%) from other congeners known from the mainland of India and Myanmar. The new species also show substantial genetic distance (13.13% to 16.44%) to other congeners distributed in Sumatra. Both ML and BA phylogenetic trees showed similar topologies with high posterior probabilities and bootstrap supports (Fig. 4, Supplementary Fig. S4) and showed that the three *Crocidura* species from the AN archipelago included in the analysis do not form a monophyletic group but are related to entirely different branches of the evolutionary tree.

## Discussion

Island ecosystems are regarded as discrete biogeographic units and as a significant model for evolutionary studies^8,32,64^. In the Miocene–Pliocene, volcanic eruption produced many new islands and their sporadic land connections during the Pleistocene, allowed both geographic and temporal processes of species diversification in Southeast Asia^32^. The Indian plate separated from Africa-Madagascar- Seychelles and drifted towards the Eurasian plate, which also allowed multiple opportunities for animal oversea dispersal and biological connections between the India mainland and Southeast Asia^65^. Due to the remoteness and inaccessibility throughout the year, the smaller islands of the AN archipelago and other tropical islands are little explored in comparison to larger islands.

The political boundaries of India include a number of islands in both the Arabian Sea and the Bay of Bengal. The islands of the Arabian Sea are primarily built up by coral reefs, whereas the Bay of Bengal islands are distinguished by habitable submarine mountains^45^. Narcondam Island is one of 836 islands of the AN Archipelago, and is a small dormant volcanic island with almost 80% forest cover^45,66^ (Fig. 2). It is believed that the volcano was active during Holocene eruptions^66,67^. However, bathymetric study revealed that a number of seamounts have been well-developed on the Andaman seafloor, which changes the trend of the volcanic arc from northeast to southeast to Sumatra Island. Hence, it is evidenced that there is a direct connection between the submerged volcanic arc-chain of the Andaman Sea from Sumatra to Barren and Narcondam volcanic islands^68,69^. The island has been recognised by UNESCO as a World Heritage site due to its sensitive ecosystem and the occurrence of an endangered species, the Narcondam hornbill (*Rhyticeros narcondami*). Further, this island has been also designated as Wildlife Sanctuary under the provisions of the Indian Wildlife (Protection) Act, 1972.

The unparalleled biogeography of oceanic islands provides a suitable habitat for many *Crocidura* species discovered in the recent past. The known distribution of the new shrew species is restricted to Narcondam Island, a very small island. Isolated islands appear to provide a suitable habitat for many endemic *Crocidura* species, such as *C. canariensis*, which is restricted to the Canary Islands, *C. fingui* and *C. thomensis*, restricted to the Sao Tome and Principe Islands, *C. orii*, restricted to the Amamioshima group of the Ryukyu Islands, and *C. trichura*, restricted to Christmas Island^4,70^. The present discovery of a new shrew from isolated Narcondam Island adds an interesting detail to this pattern.

Considering the molecular-based species identification, the new species is clearly genetically distinct. Previous studies already showed that the mtCytb gene can often be effectively used to discriminate shrew species and to detect cryptic diversity in different geographical regions^7,42,43,57^. The genetic assessment of *Crocidura* species also facilitates the description of their radiation and diversification in Southeast Asian countries^32^. The estimated K2P genetic distance, ML and BA phylogenies clearly discriminate all the studied shrew species with sufficient genetic distances and distinct clustering. The new species *C. narcondamica* sp. nov. shows a substantial genetic distance (12.02%) to *C. rapax* (distributed in China, India, Myanmar, and Taiwan), and even larger distances in comparison with other AN archipelago species such as *C. andamanensis* with 16.61%, and *C. nicobarica* with 15.09%. The initial phylogenetic analysis indicates that the numerous endemic *Crocidura* species are not the result of a local radiation, as could be initially expected, but appear to be derived from at least three independent colonization events. To understand this surprising pattern, we recommend generating more molecular data of this group of mammals from different geographical regions to clarify their in-depth phylogenetic relationships, provide estimates of the divergence events, and allow a better alignment with the biogeographical history of the Indo-Malayan and Sundaic realms.

So far, Narcondam Island is popularly known by the occurrence of the endemic Narcondam hornbill. The ecological study of animals on this island was initially restricted only to this bird. Later on, researchers focused on a faunal expedition and recorded 17 fishes, 8 reptiles, 28 birds, 2 mammals (Chinese Forest Rat, *Rattus andamanensis* and Island Flying Fox, *Pteropus hypomelanus*), 13 spiders, 8 butterflies, and 2 sea cucumbers from this isolated island^47^. However, the known diversity of the mammalian fauna is very sparse on Narcondam Island; especially because no data were reported on soricid fauna (shrews). The discovery of *C. jenkinsi* on South Andaman Island by Chakraborty^12^ was the latest discovery of a *Crocidura* species from India. With this new description of the Narcondam shrew, altogether twelve species of *Crocidura* are now known from India including the AN Archipelago; viz., *C. andamanensis*, *C. attenuata*, *C. fuliginosa*, *C. hispida*, *C. horsfieldii*, *C. jenkinsi*, *C. nicobarica*, *C. pergrisea*, *C. pullata*, *C. rapax*, *C. vorax*, and *C. narcondamica* sp. nov. Among the Indian *Crocidura* shrews, four species known from the AN Archipelago have been categorized as threatened by the IUCN Red List of Threatened Species (2020-3) due to their remarkable endemism^70^. Habitat loss due to selective logging, anthropogenic activities, and natural disasters have been identified as the major threats for this group of animals in the AN Archipelago^71,72^. As the Narcondam Island is uninhabited, the new species *C. narcondamica* sp. nov. may not face the anthropogenic disturbances, but the extremely restricted insular habitat and the associated limited population size will automatically result in increased vulnerability of the species^73^. Raptors may be assumed to be potential natural predators of this species, but this threat is probably negligible compared to the generally precarious position of any small-island endemic with respect to natural disasters and stochastic population fluctuations. Further studies on the taxonomy, ecology, and distribution of the new species will help to understand its present status in more detail.

## Supplementary Information

Supplementary Information.

## Data Availability

The following information was supplied regarding the accessibility of DNA sequences: The generated partial fragment of mitochondrial Cytochrome b gene sequences are deposited in GenBank of NCBI under accession number MW417367 and MW417368.
