# Supplementary material for: Discovery of a new mammal species (Soricidae: Eulipotyphla) from Narcondam volcanic island, India
Source: Sci Rep. 2021 May 3;11:9416. doi: 10.1038/s41598-021-88859-4 (PMC8093265; doi:10.1038/s41598-021-88859-4)
Supplement: Supplementary file 1 — Supplementary Information. [file 41598_2021_88859_MOESM1_ESM.docx]

**Supplementary Information**

**Discovery of a new mammal species (Soricidae: Eulipotyphla) from Narcondam volcanic island, India**

Manokaran Kamalakannan^1^*, Chandrakasan Sivaperuman^3^, Shantanu Kundu^2^*, Govindarasu Gokulakrishnan^3^, Chinnadurai Venkatraman^1^ & Kailash Chandra^1,2,3^

1. *Mammal and Osteology Section, Zoological Survey of India, Kolkata-700053, India.*
2. *Centre for DNA Taxonomy, Molecular Systematics Division, Zoological Survey of India, Kolkata-700053, India.*
3. *Andaman and Nicobar Regional Centre, Zoological Survey of India, Port Blair- 744102, India.*

**Corresponding author’s Email: kamalakannanm1@gmail.com, shantanu1984@gmail.com*

**Figure S1.** Ventral view of cranium showing (**a**) 4 upper unicuspids in genus *Suncus* (*S. murinus*, ZSI 16523) and (**b**) 3 unicuspids in *Crocidura* (*C. attenuata*, ZSI 16129). The photographs were captured by the first author using a Nikon D7000 camera and edited manually in Adobe Photoshop CS 8.0.


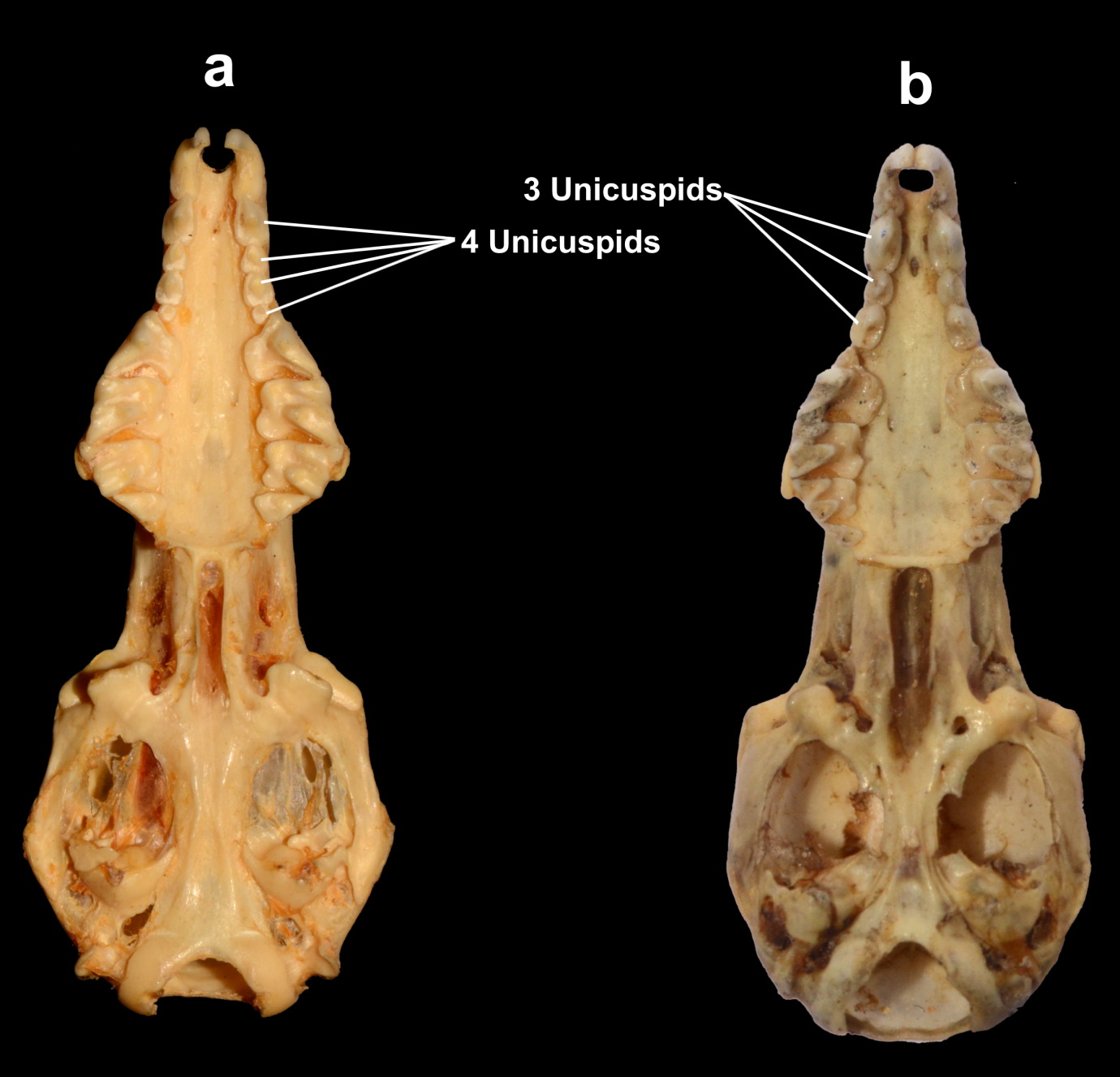


**Figure S2.** *Crocidura attenuata* (ZSI-16129, male). Views of stuffed skin (**a**) dorsal and (**b**) lateral. Views of the cranium (**c**) dorsal, (**d**) ventral and (**e**) lateral. Views of the mandible (**f**) occlusal and (**g**) lateral. (**BC**= Braincase; **LR**= lambdoidal ridge). The photographs were captured by the first author using a Nikon D7000 camera and edited manually in Adobe Photoshop CS 8.0.


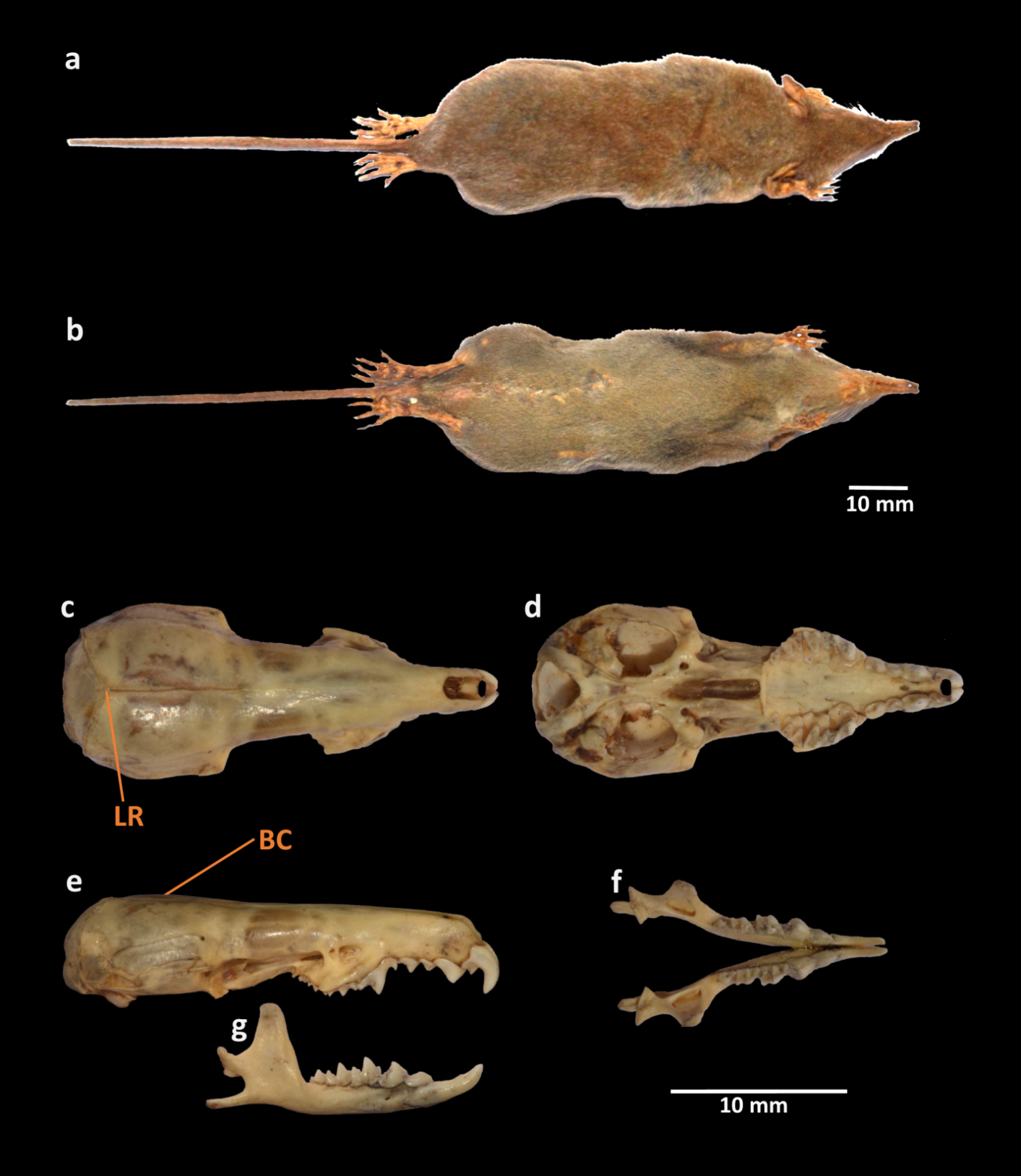


**
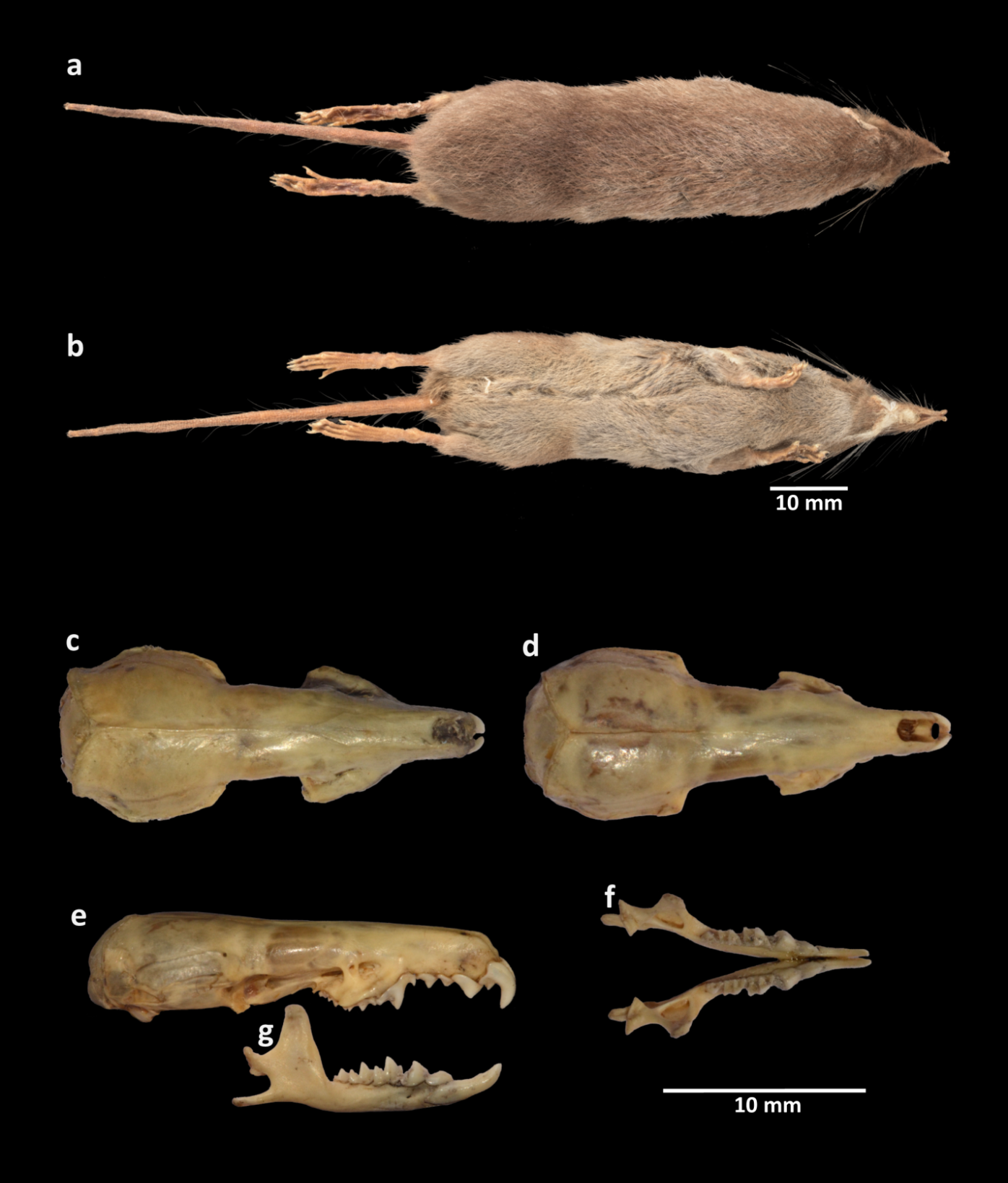
Figure S3.** *Crocidura jenkinsi* (ZSI-19860, female). Views of stuffed skin (**a**) dorsal and (**b**) lateral. Views of the cranium (**c**) dorsal, (**d**) ventral and (**e**) lateral. Views of the mandible (**f**) occlusal and (**g**) lateral. The photographs were captured by the first author using a Nikon D7000 camera and edited manually in Adobe Photoshop CS 8.0.


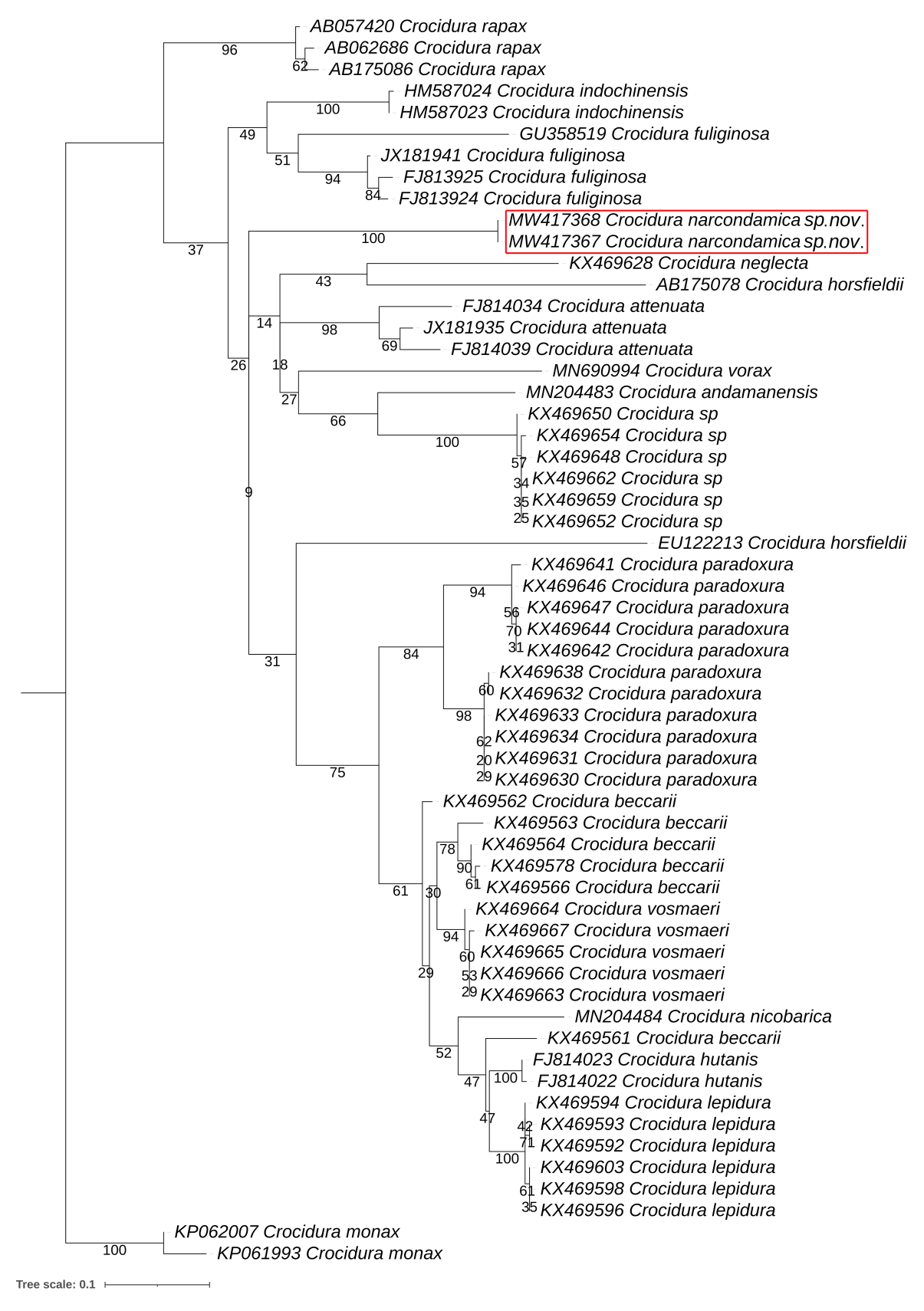
**Figure S4.** The Maximum-Likelihood phylogenetic analysis of mitochondrial Cytb gene showed distinct clustering of *C. narcondamica* sp. nov. in compararion with other *Crocidura* species distributed in the AN Archipelago, the mainland of India, Myanmar, and from Sumatra. The bootstrap supports were noted with each node. The GenBank accession numbers and species name were marked as per clade pattern. The distinct clade of the newly discovered species was marked by red colour box. The figure prepared in web-based iTOL tool (https://itol.embl.de/) and edited manually in Adobe Photoshop CS 8.0.

**Table S1.** List of white-toothed shrew *Crocidura* with their authority distributed in the Indian mainland, the AN Archipelago, Myanmar, and from Sumatra with their elsewhere distribution, IUCN status, and comparison approaches.

| **Sl. No.** | **Species** | **Distribution** | **IUCN status** | **Comparison** |
| --- | --- | --- | --- | --- |
| 1 | *Crocidura andamanensis*  Miller, 1902 | Andaman and Nicobar Archipelago | Critically Endangered | Both morphology and molecular |
| 2 | *C. attenuata*  Milne-Edwards, 1872 | Cambodia, China, India, Laos, Malaysia, Myanmar, Philippines, Taiwan, Thailand, and Viet Nam | Least Concerned | Both morphology and molecular |
| 3 | *C. cranbrooki*  Jenkins et al. 2009 | Myanmar | Not Assessed | Morphology |
| 4 | *C. fuliginosa*  (Blyth, 1855) | Cambodia, China, India, Laos, Malaysia, Myanmar, Thailand, and Viet Nam | Least Concerned | Both morphology and molecular |
| 5 | *C. hispida*  Thomas, 1913 | Andaman and Nicobar Archipelago | Vulnerable | Morphology |
| 6 | *C. horsfieldii*  (Tomes, 1856) | India, Nepal, Sri Lanka, and Thailand | Data Deficient | Both morphology and molecular |
| 7 | *C. indochinensis*  Robinson & Kloss, 1922 | China, Laos Republic, Myanmar, Thailand, and Viet Nam | Least Concerned | Both morphology and molecular |
| 8 | *C. jenkinsi*  Chakraborty, 1978 | Andaman and Nicobar Archipelago | Critically Endangered | Morphology |
| 9 | *C. nicobarica*  Miller, 1902 | Andaman and Nicobar Archipelago | Critically Endangered | Both morphology and molecular |
| 10 | *C. pergrisea*  Miller, 1913 | India and Pakistan | Data Deficient | Morphology |
| 11 | *C. pullata*  Miller, 1911 | India and Pakistan | Data Deficient | Morphology |
| 12 | *C. rapax*  G. Allen, 1923 | China, India, Myanmar, and Taiwan | Data Deficient | Both morphology and molecular |
| 13 | *C. vorax*  G. Allen, 1923 | China, India, Laos, Thailand, and Viet Nam | Least Concerned | Both morphology and molecular |
| 14 | *C. hutanis*  Ruedi & Vogel, 1995 | Sumatra, Indonesia | Least Concerned | Molecular |
| 15 | *C. beccarii*  Dobson, 1887 | Sumatra, Indonesia | Least Concerned | Molecular |
| 16 | *C. paradoxura*  Dobson, 1886 | Sumatra, Indonesia | Least Concerned | Molecular |
| 17 | *C. lepidura*  Lyon, 1908 | Sumatra, Indonesia | Least Concerned | Molecular |
| 18 | *C. neglecta*  Jentink, 1888 | Sumatra, Indonesia | Not Assessed | Molecular |
| 19 | *C. vosmaeri*  Jentink, 1888 | Bangka Island, Sumatra, Indonesia | Data Deficient | Molecular |
| 20 | *Crocidura* sp.  Demos et al. 2016 | Sumatra, Indonesia | Not Assessed | Molecular |

**Table S2.** External and craniodental measurements of the examined specimens of *C. narcondamica* sp. nov. (holotype and paratype) and its congeners are known from the Andaman and Nicobar Archipelago, the mainland of India and from Myanmar. For trait abbreviations (columns) see Material and Methods. All measurements are in millimetres with Mean ± SD, range, and sample size within first bracket. Species abbreviation: CNA= *C. narcondamica* sp. nov.; CJI= *C. jenkinsi*; CNI= *C. nicobarica*; CAS= *C. andamanensis*; CHA= *C.* *hispida*; CCI= *C. cranbrooki*; CIS= *C. indochinensis*; CAA= *C. attenuata*; CFA= *C. fuliginosa*; CHI= *C. horsfieldii*; CPA= *C. pullata*; CRX=  *C. rapax*; CVX= *C. vorax*; CPE= *C. pergrisea*.

| **Variable** | **CNA**  **Holotype** | **CNA**  **Paratype** | **CJI** | **CNI** | **CAS** | **CHA** | **CCI** | **CIS** | **CAA**  **(40)** | **CFA**  **(73)** | **CHI**  **(13)** | **CPA**  **(20)** | **CRX**  **(10)** | **CVX**  **(12)** | **CPE**  **(86)** |
| --- | --- | --- | --- | --- | --- | --- | --- | --- | --- | --- | --- | --- | --- | --- | --- |
| **Source** | *C. narcondamica* sp. nov. | | ZSI 19860 | Miller 1912 | | Menon 2014 | Jenkins et al. 2009 | | Jiang and Hoffmann 2001 | | | | | | Tez and Kefelioglu 2000 |
| **HB** | 67 | 63 | 107 | 120 | 114 | 85 | 76.13 ± 7.77  65–86 (8) | 60.5 ± 6.35  53–71 (6) | 76.0 ± 6.60  60.0–89.0 | 86.3 ± 6.7  72.0–100.0 | 61.1 ± 6.18  49.0–71.0 | 83.2 ± 4.00  73.0–89.0 | 64.1 ± 4.51  56.0–70.0 | 67.3 ±9.77  54.0–90 | 73.05±5.30  65-86 |
| **TL** | 58.5 | 55.6 | 95.2 | 90 | 86 | 103 | 74.13 ± 7.16  65–88 (8) | 45.3 ± 3.77  40–50 (6) | 50.5 ± 5.29  43.0–60.0 | 74.1 ±5.4  62.0–89.0 | 40.8 ± 5.97  30.0–48.0 | 44.2 ± 4.38  39.0–53.0 | 42.4 ± 2.80  38.0–47.0 | 46.7 ±3.34  41.0–51.0 | 43.56±2.75  37-51 |
| **HF** | 13.4 | 12.4 | 26 | 24 | 25 |  | 14.94 ± 0.64  14–16 (9) | 11.53 ± 1.24  10–13 (6) | 13.4 ± 1.12  11.0–16.0 | 16.5 ± 1.1  15.0–19.0 | 11.0 ±1.04  10.0–13.0 | 15.06 ± 0.66  14.0–16.0 | 11.9 ± 0.60  11.0–13.0 | 12.3 ± 0.78  11.0–14.0 | 13.73±0.68  13-15 |
| **CIL** | 19.6 | 18.9 | 27.6 | 29 | 25.6 |  | 21.38 ±0.67  19.9–22.2 (14) | 17.31 ±0.21  17.0–17.6 (6) | 20.13 ± 0.70  18.51–21.49 | 23.26 ± 0.64  22.04–25.00 | 17.18 ±0.40  16.14–17.82 | 20.26 ± 0.46  19.45–21.21 | 18.14 ± 0.27  17.45–18.33 | 19.09 ± 0.77  17.74–20.11 | 17.97±0.59  16.30-19.30 |
| **BB** | 8.7 | 8 | 11.4 |  |  |  | 9.31 ± 0.2  9.0–9.6 (14) | 8.11 ± 0.22  7.8–8.4 (6) | 9.02 ± 0.32  8.32–9.82 | 10.33 ± 0.35  9.40–11.19 | 8.17 ± 0.25  7.69–8.61 | 9.27 ± 0.20  8.92–9.63 | 8.32 ±0.35  7.64–8.78 | 8.40 ± 0.48  7.57–9.06 | 8.68±0.38  8.05-9.95 |
| **UTR** | 8.8 | 7.9 | 12.5 | 13 | 12 |  | 9.29 ± 0.29  8.6–9.8 (17) | 7.24 ± 0.13  7.1–7.5 (7) | 8.50 ± 0.28  8.07–9.10 | 10.06 ± 0.35  9.21–10.85 | 7.07 ±0.33  6.46–7.72 | 8.61 ± 0.22  8.22–9.00 | 7.57 ±0.30  7.11–8.07 | 7.77 ± 0.39  7.16–8.23 | 8.18±0.26  7.45-8.90 |
| **MTR** | 7.7 | 7 | 11 | 12 | 11 |  | 8.68 ± 0.28  8.0–9.2 (16) | 6.73 ± 0.11  6.6–6.9 (7) | 8.11 ±0.28  7.36–8.70 | 9.51 ± 0.28  8.74–10.33 | 6.83 ±0.18  6.58–7.26 | 8.94 ± 0.22  7.77–8.70 | 7.25 ±0.27  6.87–7.79 | 7.37 ± 0.32  6.79–7.86 | 7.33±0.37  6.20-8.05 |
| **ML** | 11.6 | 10.8 | 17.6 | 15 | 15 |  | 13.35 ± 0.45  12.2–13.9 (16) | 10.45 ± 0.23  10.2–10.8 (7) | 10.77 ± 0.44  9.68–11.55 | 12.61 ± 0.41  11.74–13.45 | 9.18 ±0.26  8.78–9.85 | 10.59 ± 0.26  10.12–11.09 | 9.58 ± 0.39  8.70–10.13 | 9.60 ± 0.37  8.77–10.12 | 10.14±0.34  9.30-10.85 |

**Table S3.** List of the *Crocidura* species, collection localities, and GenBank accession numbers for all specimens (generated and GenBank) used in this study.

| **Sl. No.** | **Species** | **Collection locality** | **Accession number** |
| --- | --- | --- | --- |
| 1 | *Crocidura beccarii* | Mt. Singgalang, Sumatra, Indonesia | KX469561 |
| 2 | *Crocidura beccarii* | Mt. Singgalang, Sumatra, Indonesia | KX469562 |
| 3 | *Crocidura beccarii* | Mt. Singgalang, Sumatra, Indonesia | KX469563 |
| 4 | *Crocidura beccarii* | Mt. Tujuh, Sumatra, Indonesia | KX469564 |
| 5 | *Crocidura beccarii* | Mt. Tujuh, Sumatra, Indonesia | KX469566 |
| 6 | *Crocidura beccarii* | Mt. Tujuh, Sumatra, Indonesia | KX469578 |
| 7 | *Crocidura hutanis* | Gunung Leuser NP, Sumatra, Indonesia | FJ814022 |
| 8 | *Crocidura hutanis* | Gunung Leuser NP, Sumatra, Indonesia | FJ814023 |
| 9 | *Crocidura lepidura* | Mt. Tujuh, Sumatra, Indonesia | KX469592 |
| 10 | *Crocidura lepidura* | Mt. Tujuh, Sumatra, Indonesia | KX469593 |
| 11 | *Crocidura lepidura* | Mt. Tujuh, Sumatra, Indonesia | KX469594 |
| 12 | *Crocidura lepidura* | Mt. Tujuh, Sumatra, Indonesia | KX469596 |
| 13 | *Crocidura lepidura* | Mt. Tujuh, Sumatra, Indonesia | KX469598 |
| 14 | *Crocidura lepidura* | Mt. Tujuh, Sumatra, Indonesia | KX469603 |
| 15 | *Crocidura monax* | Mt. Kilimanjaro, Tanzania | KP061993 |
| 16 | *Crocidura monax* | North Pare Mts., Tanzania | KP062007 |
| 17 | *Crocidura neglecta* | Mt. Tujuh, Sumatra, Indonesia | KX469628 |
| 18 | *Crocidura paradoxura* | Mt. Singgalang, Sumatra, Indonesia | KX469630 |
| 19 | *Crocidura paradoxura* | Mt. Singgalang, Sumatra, Indonesia | KX469631 |
| 20 | *Crocidura paradoxura* | Mt. Singgalang, Sumatra, Indonesia | KX469632 |
| 21 | *Crocidura paradoxura* | Mt. Singgalang, Sumatra, Indonesia | KX469633 |
| 22 | *Crocidura paradoxura* | Mt. Singgalang, Sumatra, Indonesia | KX469634 |
| 23 | *Crocidura paradoxura* | Mt. Singgalang, Sumatra, Indonesia | KX469638 |
| 24 | *Crocidura paradoxura* | Mt. Tujuh, Sumatra, Indonesia | KX469641 |
| 25 | *Crocidura paradoxura* | Mt. Tujuh, Sumatra, Indonesia | KX469642 |
| 26 | *Crocidura paradoxura* | Mt. Tujuh, Sumatra, Indonesia | KX469644 |
| 27 | *Crocidura paradoxura* | Mt. Tujuh, Sumatra, Indonesia | KX469646 |
| 28 | *Crocidura paradoxura* | Mt. Tujuh, Sumatra, Indonesia | KX469647 |
| 29 | *Crocidura* sp. nov. 2 | Mt. Singgalang, Sumatra, Indonesia | KX469648 |
| 30 | *Crocidura* sp. nov. 2 | Mt. Singgalang, Sumatra, Indonesia | KX469650 |
| 31 | *Crocidura* sp. nov. 2 | Mt. Singgalang, Sumatra, Indonesia | KX469652 |
| 32 | *Crocidura* sp. nov. 2 | Mt. Singgalang, Sumatra, Indonesia | KX469654 |
| 33 | *Crocidura* sp. nov. 2 | Mt. Singgalang, Sumatra, Indonesia | KX469659 |
| 34 | *Crocidura* sp. nov. 2 | Mt. Singgalang, Sumatra, Indonesia | KX469662 |
| 35 | *Crocidura vosmaeri* | Bangka Island, Sumatra, Indonesia | KX469664 |
| 36 | *Crocidura vosmaeri* | Bangka Island, Sumatra, Indonesia | KX469665 |
| 37 | *Crocidura vosmaeri* | Bangka Island, Sumatra, Indonesia | KX469663 |
| 38 | *Crocidura vosmaeri* | Bangka Island, Sumatra, Indonesia | KX469666 |
| 39 | *Crocidura vosmaeri* | Bangka Island, Sumatra, Indonesia | KX469667 |
| 40 | *Crocidura narcondamica* sp. nov. | Narcondam Island, India | MW417367 |
| 41 | *Crocidura narcondamica* sp. nov. | Narcondam Island, India | MW417368 |
| 42 | *Crocidura horsfeildii* | Sri Lanka | EU122213 |
| 43 | *Crocidura horsfeildii* | Kanchana Buri, Sai Yok, Thailand | AB175078 |
| 44 | *Crocidura vorax* | Jinfo, Chongqing, China | MN690994 |
| 45 | *Crocidura andamanensis* | Wrightmyo, South Andaman Island, India | MN204483 |
| 46 | *Crocidura nicobarica* | Galathea, Great Nicobar Island, India | MN204484 |
| 47 | *Crocidura attenuata* | Hunan, China | FJ814034 |
| 48 | *Crocidura attenuata* | Guangxi, China | FJ814039 |
| 49 | *Crocidura attenuata* | Cat Ba, Viet Nam | JX181935 |
| 50 | *Crocidura rapax* | Nantou Co., Taiwan | AB175086 |
| 51 | *Crocidura rapax* | Nantou Xian, Xitou, Taiwan | AB062686 |
| 52 | *Crocidura rapax* | Nantou Xian, Xitou, Taiwan | AB057420 |
| 53 | *Crocidura indochinensis* | Lam Dong Province, Viet Nam | HM587024 |
| 54 | *Crocidura indochinensis* | Lam Dong Province, Viet Nam | HM587023 |
| 55 | *Crocidura fuliginosa* | Con Dao, Viet Nam | JX181941 |
| 56 | *Crocidura fuliginosa* | Pahang, Malaysia | FJ813925 |
| 57 | *Crocidura fuliginosa* | Peninsular Malaysia | FJ813924 |
| 58 | *Crocidura fuliginosa* | Vietnam | GU358519 |

**Table S4.** The between and within group Kimura-2-parameter (K2P) genetic distances of the Soricid genera (top) and *Crocidura* species (bottom) based on partial mtCytb gene in the present dataset. n/c= not calculated due to single sequence.

| ***Crocidura***  **species** | **Between species** | | | | | | | | | | | | | | | **Within species** |
| --- | --- | --- | --- | --- | --- | --- | --- | --- | --- | --- | --- | --- | --- | --- | --- | --- |
| *C. narcondamica* | 0 |  |  |  |  |  |  |  |  |  |  |  |  |  |  | 0 |
| *C. rapax* | 12.02 |  |  |  |  |  |  |  |  |  |  |  |  |  |  | 1.34 |
| *C. indochinensis* | 12.41 | 13.57 |  |  |  |  |  |  |  |  |  |  |  |  |  | 0 |
| *C. attenuata* | 12.54 | 13.39 | 12.51 |  |  |  |  |  |  |  |  |  |  |  |  | 4.24 |
| *C. fuliginosa* | 12.91 | 13.93 | 10.00 | 12.61 |  |  |  |  |  |  |  |  |  |  |  | 5.81 |
| *C. paradoxura* | 13.13 | 13.49 | 14.46 | 14.44 | 11.81 |  |  |  |  |  |  |  |  |  |  | 3.07 |
| *C. hutanis* | 13.59 | 15.21 | 13.30 | 14.23 | 13.25 | 9.60 |  |  |  |  |  |  |  |  |  | 0.25 |
| *C. beccarii* | 13.78 | 14.35 | 12.16 | 13.65 | 12.22 | 8.59 | 5.86 |  |  |  |  |  |  |  |  | 3.29 |
| *C. horsfieldii* | 15.15 | 16.90 | 15.94 | 15.48 | 15.57 | 14.75 | 14.53 | 14.69 |  |  |  |  |  |  |  | 12.96 |
| *C. nicobarica* | 15.09 | 15.00 | 14.07 | 14.32 | 13.45 | 9.08 | 5.91 | 6.11 | 14.53 |  |  |  |  |  |  | n/c |
| *C. vosmaeri* | 15.43 | 15.34 | 12.79 | 14.53 | 12.37 | 8.48 | 5.52 | 3.63 | 15.37 | 5.78 |  |  |  |  |  | 0.20 |
| *C. lepidura* | 15.71 | 16.29 | 14.67 | 14.37 | 14.53 | 10.50 | 3.42 | 6.16 | 16.32 | 5.73 | 5.62 |  |  |  |  | 0.28 |
| *Crocidura* sp. | 15.83 | 13.78 | 11.77 | 13.96 | 15.55 | 14.48 | 13.73 | 13.80 | 18.80 | 12.96 | 14.25 | 14.53 |  |  |  | 0.17 |
| *C. neglecta* | 16.44 | 15.22 | 14.98 | 14.37 | 15.19 | 14.32 | 14.56 | 14.16 | 15.32 | 15.09 | 15.29 | 15.04 | 16.06 |  |  | n/c |
| *C. vorax* | 16.57 | 14.94 | 13.01 | 14.04 | 16.22 | 13.70 | 16.44 | 14.71 | 17.18 | 13.04 | 15.31 | 15.26 | 14.62 | 15.24 |  | n/c |
| *C. andamanensis* | 16.61 | 18.00 | 16.57 | 18.44 | 17.64 | 14.42 | 17.86 | 16.47 | 18.42 | 15.35 | 17.94 | 18.37 | 13.96 | 17.64 | 15.50 | n/c |
